# rs495139 in the *TYMS*-*ENOSF1* Region and Risk of Ovarian Carcinoma of Mucinous Histology

**DOI:** 10.3390/ijms19092473

**Published:** 2018-08-21

**Authors:** Linda E. Kelemen, Madalene Earp, Brooke L. Fridley, Georgia Chenevix-Trench, on behalf of Australian Ovarian Cancer Study Group, Peter A. Fasching, Matthias W. Beckmann, Arif B. Ekici, Alexander Hein, Diether Lambrechts, Sandrina Lambrechts, Els Van Nieuwenhuysen, Ignace Vergote, Mary Anne Rossing, Jennifer A. Doherty, Jenny Chang-Claude, Sabine Behrens, Kirsten B. Moysich, Rikki Cannioto, Shashikant Lele, Kunle Odunsi, Marc T. Goodman, Yurii B. Shvetsov, Pamela J. Thompson, Lynne R. Wilkens, Thilo Dörk, Natalia Antonenkova, Natalia Bogdanova, Peter Hillemanns, Ingo B. Runnebaum, Andreas du Bois, Philipp Harter, Florian Heitz, Ira Schwaab, Ralf Butzow, Liisa M. Pelttari, Heli Nevanlinna, Francesmary Modugno, Robert P. Edwards, Joseph L. Kelley, Roberta B. Ness, Beth Y. Karlan, Jenny Lester, Sandra Orsulic, Christine Walsh, Susanne K. Kjaer, Allan Jensen, Julie M. Cunningham, Robert A. Vierkant, Graham G. Giles, Fiona Bruinsma, Melissa C. Southey, Michelle A.T. Hildebrandt, Dong Liang, Karen Lu, Xifeng Wu, Thomas A. Sellers, Douglas A. Levine, Joellen M. Schildkraut, Edwin S. Iversen, Kathryn L. Terry, Daniel W. Cramer, Shelley S. Tworoger, Elizabeth M. Poole, Elisa V. Bandera, Sara H. Olson, Irene Orlow, Liv Cecilie Vestrheim Thomsen, Line Bjorge, Camilla Krakstad, Ingvild L. Tangen, Lambertus A. Kiemeney, Katja K.H. Aben, Leon F.A.G. Massuger, Anne M. van Altena, Tanja Pejovic, Yukie Bean, Melissa Kellar, Linda S. Cook, Nhu D. Le, Angela Brooks-Wilson, Jacek Gronwald, Cezary Cybulski, Anna Jakubowska, Jan Lubiński, Nicolas Wentzensen, Louise A. Brinton, Jolanta Lissowska, Estrid Hogdall, Svend Aage Engelholm, Claus Hogdall, Lene Lundvall, Lotte Nedergaard, Paul D.P. Pharoah, Ed Dicks, Honglin Song, Jonathan P. Tyrer, Iain McNeish, Nadeem Siddiqui, Karen Carty, Rosalind Glasspool, James Paul, Ian G. Campbell, Diana Eccles, Alice S. Whittemore, Valerie McGuire, Joseph H. Rothstein, Weiva Sieh, Steven A. Narod, Catherine M. Phelan, John R. McLaughlin, Harvey A. Risch, Hoda Anton-Culver, Argyrios Ziogas, Usha Menon, Simon A. Gayther, Aleksandra Gentry-Maharaj, Susan J. Ramus, Anna H. Wu, Celeste Leigh Pearce, Alice W. Lee, Malcolm C. Pike, Jolanta Kupryjanczyk, Agnieszka Podgorska, Joanna Plisiecka-Halasa, Wlodzimierz Sawicki, Ellen L. Goode, Andrew Berchuck, Ovarian Cancer Association Consortium

**Affiliations:** 1 Hollings Cancer Center, Medical University of South Carolina, Charleston, SC 29425, USA; 2 Department of Public Health Sciences, College of Medicine, Medical University of South Carolina, Charleston, SC 29425, USA; 3 Department of Health Sciences Research, Division of Epidemiology, Mayo Clinic, Rochester, MN 55905, USA; madalene.earp@ucalgary.ca (M.E.); egoode@mayo.edu (E.L.G.); 4 Department of Biostatistics and Bioinformatics, H. Lee Moffitt Cancer Center and Research Institute, Tampa, FL 33612 USA; brooke.fridley@moffitt.org; 5 Department of Genetics and Computational Biology, QIMR Berghofer Medical Research Institute, Brisbane, QLD 4006, Australia; georgia.trench@qimr.edu.au; 6 Peter MacCallum Cancer Centre, Melbourne, VIC 3000, Australia; 7 Department of Gynecology and Obstetrics, University Hospital Erlangen, Friedrich-Alexander University Erlangen-Nuremberg, Comprehensive Cancer Center, 91054 Erlangen, Germany; peter.fasching@uk-erlangen.de (P.A.F.); fk-direktion@uk-erlangen.de (M.W.B.); alexander.hein@uk-erlangen.de (A.H.); 8 Department of Medicine, Division of Hematology and Oncology, David Geffen School of Medicine, University of California at Los Angeles, Los Angeles, CA 90095, USA; 9 Institute of Human Genetics, Friedrich-Alexander-University Erlangen-Nuremberg, Comprehensive Cancer Center Erlangen Nuremberg, Erlangen 91054, Germany; arif.ekici@uk-erlangen.de; 10 Vesalius Research Center, University of Leuven, Leuven 3001, Belgium; diether.lambrechts@vib-kuleuven.be; 11 Laboratory for Translational Genetics, Department of Oncology, University of Leuven, Leuven 3000, Belgium; 12 Division of Gynecologic Oncology, Department of Obstetrics and Gynaecology and Leuven Cancer Institute, University Hospitals Leuven, Leuven 3000, Belgium; sandrina.lambrechts@mumc.nl (S.L.); els.vannieuwenhuysen@uzleuven.be (E.V.N.); ignace.vergote@uzleuven.be (I.V.); 13 Program in Epidemiology, Division of Public Health Sciences, Fred Hutchinson Cancer Research Center, Seattle, WA 98109, USA; mrossing@fhcrc.org; 14 Department of Epidemiology, University of Washington, Seattle, WA 98402, USA; 15 Huntsman Cancer Institute, Department of Population Health Sciences, University of Utah, Salt Lake City, UT 84112, USA; jennifer.doherty@hci.utah.edu; 16 Division of Cancer Epidemiology, German Cancer Research Center (DKFZ), Heidelberg 69120, Germany; j.chang-claude@dkfz-heidelberg.de (J.C.-C.); s.behrens@dkfz.de (S.B.); 17 University Cancer Center Hamburg (UCCH), University Medical Center Hamburg-Eppendorf, 20246 Hamburg, Germany; 18 Department of Cancer Prevention and Control, Roswell Park Cancer Institute, Buffalo, NY 14263, USA; moysich@roswellpark.org (K.B.M.); rikki.cannioto@roswellpark.org (R.C.); 19 Department of Gynecological Oncology, Roswell Park Cancer Institute, Buffalo, NY 14263, USA; shashi.lele@roswellpark.org (S.L.); kunle.odunsi@roswellpark.org (K.O.); 20 Department of Cancer Prevention and Control, Samuel Oschin Comprehensive Cancer Institute, Cedars-Sinai Medical Center, Los Angeles, CA 90048, USA; marc.goodman@cshs.org (M.T.G.); pamela.thompson@cshs.org (P.J.T.); 21 Community and Population Health Research Institute, Department of Biomedical Sciences, Cedars-Sinai Medical Center, Los Angeles, CA 90048, USA; 22 Cancer Epidemiology Program, University of Hawaii Cancer Center, Honolulu, HI 96813, USA; yshvetso@cc.hawaii.edu (Y.B.S.); lynne@cc.hawaii.edu (L.R.W.); 23 Gynaecology Research Unit, Hannover Medical School, Hannover 30625, Germany; doerk.thilo@mh-hannover.de (T.D.); bogdanova.natalia@mh-hannover.de (N.B.); 24 Byelorussian Institute for Oncology and Medical Radiology Aleksandrov N.N., Minsk 223040, Belarus; antonenkova.natalia.omd@tut.by; 25 Clinics of Obstetrics and Gynaecology, Hannover Medical School, Hannover 30625, Germany; hillemanns.peter@mh-hannover.de; 26 Department of Gynecology, Jena University Hospital-Friedrich Schiller University, Jena 07743, Germany; ingo.runnebaum@med.uni-jena.de; 27 Department of Gynecology and Gynecologic Oncology, Kliniken Essen-Mitte (KEM), Essen 45136, Germany; prof.dubois@googlemail.com (A.d.B.); p.harter@gmx.de (P.H.); florian.heitz@gmx.net (F.H.); 28 Department of Gynecology and Gynecologic Oncology, Dr. Horst Schmidt Kliniken Wiesbaden, Wiesbaden 65199, Germany; 29 Praxis für Humangenetik, Wiesbaden 65187, Germany; iraschwaab@gmx.de; 30 Department of Pathology, University of Helsinki and Helsinki University Hospital, Helsinki 00290, Finland; ralf.butzow@hus.fi; 31 Department of Obstetrics and Gynecology, University of Helsinki and Helsinki University Hospital, Helsinki 00290, Finland; liisa.pelttari@helsinki.fi (L.M.P.); heli.nevanlinna@hus.fi (H.N.); 32 Division of Gynecologic Oncology, Department of Obstetrics, Gynecology and Reproductive Sciences, University of Pittsburgh School of Medicine, Pittsburgh, PA 15213, USA; fm@cs.cmu.edu (F.M.); redwards@mail.magee.edu (R.P.E.); jkelley@mail.magee.edu (J.L.K.); 33 Department of Epidemiology, University of Pittsburgh Graduate School of Public Health, Pittsburgh, PA 15213, USA; 34 Women’s Cancer Research Program, Magee-Women's Research Institute and Hillman Cancer Center, Pittsburgh, PA 15213, USA; 35 School of Public Health, The University of Texas Health Science Center at Houston (UTHealth), Houston, TX 77030, USA; roberta.b.ness@uth.tmc.edu; 36 Women’s Cancer Program at the Samuel Oschin Comprehensive Cancer Institute, Cedars-Sinai Medical Center, Los Angeles, CA 90048, USA; beth.karlan@cshs.org (B.Y.K.); jenny.lester@cshs.org (J.L.); sandra.orsulic@cshs.org (S.O.); christine.walsh@cshs.org (C.W.); 37 Department of Gynaecology, Rigshospitalet, University of Copenhagen, DK-2100 Copenhagen, Denmark; susanne@cancer.dk (S.K.K.); claus.hogdall@regionh.dk (C.H.); lene.lundvall@regionh.dk (L.L.); 38 Department of Virus, Lifestyle and Genes, Danish Cancer Society Research Centre, DK-2100 Copenhagen, Denmark; allan@cancer.dk (A.J.); hogdall@dadlnet.dk (E.H.); 39 Department of Laboratory Medicine and Pathology, Division of Experimental Pathology, Mayo Clinic, Rochester, MN 55905, USA; cunningham.julie@mayo.edu; 40 Department of Health Sciences Research, Division of Biomedical Statistics and Informatics, Mayo Clinic, Rochester, MN 55905, USA; vierkant.robert@mayo.edu; 41 Centre for Epidemiology and Biostatistics, University of Melbourne, VIC 3010, Australia; graham.giles@cancervic.org.au; 42 Cancer Epidemiology and Intelligence Division, Cancer Council Victoria, Melbourne, VIC 3004, Australia; fiona.bruinsma@cancervic.org.au; 43 Department of Epidemiology and Preventive Medicine, Monash University, Melbourne, VIC 3800, Australia; 44 Department of Pathology, University of Melbourne, Melbourne, VIC 3010, Australia; msouthey@unimelb.edu.au (M.C.S.); ian.campbell@petermac.org (I.G.C.); 45 Department of Epidemiology, The University of Texas MD Anderson Cancer Center, Houston, TX 77030, USA; mhildebr@mdanderson.org (M.A.T.H.); xwu@mdanderson.org (X.W.); 46 College of Pharmacy and Health Sciences, Texas Southern University, Houston, TX 77004, USA; liang_dx@tsu.edu; 47 Department of Gynecologic Oncology, The University of Texas MD Anderson Cancer Center, Houston, TX 77030, USA; khlu@mdanderson.org; 48 Department of Cancer Epidemiology, H. Lee Moffitt Cancer Center and Research Institute, Tampa, FL 33612, USA; thomas.sellers@moffitt.org (T.A.S.); shelley.tworoger@moffitt.org (S.S.T.); ocacdata@duke.edu (C.M.P.); 49 Laura and Isaac Perlmutter Cancer Center, New York University Langone Health, New York, NY 10016, USA; Douglas.Levine@nyumc.org; 50 Department of Public Health Sciences, University of Virginia, Charlottesville, VA 22908, USA; jms2yf@virginia.edu; 51 Department of Statistical Science, Duke University, Durham, NC 27708, USA; iversen@stat.duke.edu; 52 Department of Epidemiology, Harvard T.H. Chan School of Public Health, Boston, MA 02115, USA; kterry@partners.org (K.L.T.); dcramer@partners.org (D.W.C.); 53 Obstetrics and Gynecology Epidemiology Center, Brigham and Women’s Hospital and Harvard Medical School, Boston, MA 02115, USA; 54 Channing Division of Network Medicine, Brigham and Women’s Hospital and Harvard Medical School, Boston, MA 02115, USA; nhlip@channing.harvard.edu; 55 Cancer Prevention and Control Program, Rutgers Cancer Institute of New Jersey, New Brunswick, NJ 08903, USA; banderel@cinj.rutgers.edu; 56 Memorial Sloan Kettering Cancer Center, Department of Epidemiology and Biostatistics, New York, NY 10065, USA; olsons@mskcc.org (S.H.O.); orlowi@mskcc.org (I.O.); pikem@mskcc.org (M.C.P.); 57 Department of Gynecology and Obstetrics, Haukeland University Hospital, Bergen 5021, Norway; Liv.Vestrheim@uib.no (L.C.V.T.); line.bjorge@uib.no (L.B.); camilla.krakstad@k2.uib.no (C.K.); ingvild.tangen@k2.uib.no (I.L.T.); 58 Centre for Cancer Biomarkers CCBIO, Department of Clinical Science, University of Bergen, Bergen 5020, Norway; 59 Radboud University Medical Centre, Radboud Institute for Health Sciences, Nijmegen 6525 EZ, The Netherlands; bart.kiemeney@radboudumc.nl (L.A.K.); k.aben@iknl.nl (K.K.H.A.); 60 Netherlands Comprehensive Cancer Organisation, Utrecht 3511 DT, The Netherlands; 61 Radboud University Medical Centre, Department of Obstetrics and Gynecology, Nijmegen 6525 GA, The Netherlands; leon.massuger@radboudumc.nl (L.F.A.G.M.); anne.vanaltena@radboudumc.nl (A.M.v.A.); 62 Department of Obstetrics and Gynecology, Oregon Health and Science University, Portland, OR 97239, USA; pejovict@ohsu.edu (T.P.); beany@ohsu.edu (Y.B.); kellar@ohsu.edu (M.K.); 63 Knight Cancer Institute, Oregon Health and Science University, Portland, OR 97239, USA; 64 Division of Epidemiology, Biostatistics and Preventive Medicine, Department of Internal Medicine, University of New Mexico, Albuquerque, NM 87131, USA; lcook@salud.unm.edu; 65 Cancer Control Research, British Columbia Cancer Agency, Vancouver, BC V5Z 1L3, Canada; nle@bccrc.ca; 66 Canada’s Michael Smith Genome Sciences Centre, British Columbia Cancer Agency, Vancouver, BC V5Z 1L3, Canada; abrooks-wilson@bcgsc.ca; 67 Department of Biomedical Physiology and Kinesiology, Simon Fraser University, Burnaby, BC V5A 1S6, Canada; 68 International Hereditary Cancer Center, Department of Genetics and Pathology, Pomeranian Medical University, Szczecin 71-252, Poland; jgron@sci.pam.szczecin.pl (J.G.); cezarycy@sci.pam.szczecin.pl (C.C.); aniaj@sci.pam.szczecin.pl (A.J.); lubinski@pum.edu.pl (J.L.); 69 Independent Laboratory of Molecular Biology and Genetic Diagnostics, Pomeranian Medical University, Szczecin 70-111, Poland; 70 Division of Cancer Epidemiology and Genetics, National Cancer Institute, Bethesda, MD 20892, USA; wentzenn@mail.nih.gov (N.W.); brintonl@mail.nih.gov (L.A.B.); 71 Department of Cancer Epidemiology and Prevention, M. Sklodowska-Curie Institute-Oncology Center, Warsaw 02-034, Poland; lissowsj@coi.waw.pl; 72 Department of Pathology, Herlev Hospital, University of Copenhagen, Copenhagen DK-2100, Denmark; 73 Department of Radiation Oncology, Rigshospitalet, University of Copenhagen, Copenhagen DK-2100, Denmark; svend.aage.engelholm@rh.regionh.dk; 74 Department of Pathology, Rigshospitalet, University of Copenhagen, Copenhagen DK-2100, Denmark; lotte.thomsen.01@regionh.dk; 75 The Centre for Cancer Genetic Epidemiology, Department of Public Health and Primary Care, University of Cambridge, Cambridge CB1 8RN, UK; pp10001@medschl.cam.ac.uk; 76 The Centre for Cancer Genetic Epidemiology, Department of Oncology, University of Cambridge, Cambridge CB1 8RN, UK; emd43@medschl.cam.ac.uk (E.D.); hs310@medschl.cam.ac.uk (H.S.); jpt34@medschl.cam.ac.uk (J.P.T.); 77 Ovarian Cancer Action Research Centre, Department of Surgery and Cancer, Imperial College London, London W12 0NN, UK; i.mcneish@imperial.ac.uk; 78 Department of Gynaecological Oncology, Glasgow Royal Infirmary, Glasgow G4 0SF, UK; nadeem.siddiqui@ggc.scot.nhs.uk; 79 Cancer Research UK Clinical Trials Unit, The Beatson West of Scotland Cancer Centre, Glasgow G12 0YN, UK; karen.carty@glasgow.ac.uk (K.C.); ros.glasspool@ggc.scot.nhs.uk (R.G.); james.paul@glasgow.ac.uk (J.P.); 80 Cancer Genetics Laboratory, Research Division, Peter MacCallum Cancer Centre, St Andrews Place, East Melbourne, VIC 3000, Australia; 81 Sir Peter MacCallum Department of Oncology, University of Melbourne, Melbourne, VIC 3000, Australia; 82 Faculty of Medicine, University of Southampton, Southampton SO17 1BJ, UK; d.m.eccles@soton.ac.uk; 83 Department of Health Research and Policy, Stanford University School of Medicine, Stanford, CA 94305, USA; alicesw@stanford.edu (A.S.W.); vmcguire@stanford.edu (V.M.); 84 Department of Health Science and Policy, Icahn School of Medicine at Mount Sinai, New York, NY 10029, USA; joseph.rothstein@mssm.edu (J.H.R.); weiva.sieh@mssm.edu (W.S.); 85 Department of Genetics and Genomic Sciences, Icahn School of Medicine at Mount Sinai, New York, NY 10029, USA; joseph.rothstein@mssm.edu (J.H.R.); weiva.sieh@mssm.edu (W.S.); 86 Women’s College Research Institute, University of Toronto, Toronto, ON M5S 1A8, Canada; steven.narod@wchospital.ca; 87 Public Health Ontario, Samuel Lunenfeld Research Institute, Toronto, ON M5T 3L9, Canada; jmclaugh@lunenfeld.ca; 88 Department of Chronic Disease Epidemiology, Yale School of Public Health, New Haven, CT 06510, USA; harvey.risch@yale.edu; 89 Department of Epidemiology, Genetic Epidemiology Research Institute, School of Medicine, University of California Irvine, Irvine, CA 92617, USA; hantoncu@uci.edu (H.A-C.); aziogas@uci.edu (A.Z.); 90 MRC Clinical Trials at UCL, Institute of Clinical Trials & Methodology, Population Health Sciences, University College London, London, WC1V 6LJ, UK; u.menon@ucl.ac.uk (U.M.); a.gentry-maharaj@ucl.ac.uk (A.G.-M.); 91 Department of Biomedical Sciences and Center for Cancer Prevention and Translational Genomics, Samuel Oschin Comprensive Cancer Institute, Cedars-Sinai Medical Center, Los Angeles, CA 90048, USA; simon.gayther@cshs.org; 92 School of Women’s and Children’s Health, Faculty of Medicine, University of New South Wales, Sydney, NSW 2052, Australia; s.ramus@unsw.edu.au; 93 The Kinghorn Cancer Centre, Garvan Institute of Medical Research, 384 Victoria Street, Darlinghurst, NSW 2010, Australia; 94 Department of Preventive Medicine, Keck School of Medicine, University of Southern California Norris Comprehensive Cancer Center, Los Angeles, CA 90033, USA; anna.wu@med.usc.edu (A.H.W.); lpearce@umich.edu (C.L.P.); 95 Department of Epidemiology, School of Public Health, University of Michigan, Ann Arbor, MI 48109, USA; 96 Department of Public Health, California State University, Fullerton, CA 92831, USA; alicelee@fullerton.edu; 97 Department of Pathology and Laboratory Diagnostics, Maria Sklodowska-Curie Institute-Oncology Center, Warsaw 02-034, Poland; jkupry@coi.waw.pl (J.K.); ag.podgorski@yahoo.de (A.P.); jopliha@coi.waw.pl (J.P.-H.); 98 Department of Obstetrics, Gynecology and Oncology, Second Faculty of Medicine, Medical University of Warsaw, Mazovian Bródno Hospital, Warsaw 03-242, Poland; saw55@wp.pl; 99 Department of Obstetrics and Gynecology, Duke University Medical Center, Durham, NC 27710, USA; andrew.berchuck@duke.edu

**Keywords:** consortia, enolase superfamily member 1, expression quantitative trait locus, genetics, gynecology, ovarian neoplasms, single-nucleotide polymorphism, thymidylate synthase

## Abstract

Thymidylate synthase (TYMS) is a crucial enzyme for DNA synthesis. TYMS expression is regulated by its antisense mRNA, ENOSF1. Disrupted regulation may promote uncontrolled DNA synthesis and tumor growth. We sought to replicate our previously reported association between rs495139 in the *TYMS-ENOSF1* 3′ gene region and increased risk of mucinous ovarian carcinoma (MOC) in an independent sample. Genotypes from 24,351 controls to 15,000 women with invasive OC, including 665 MOC, were available. We estimated per-allele odds ratios (OR) and 95% confidence intervals (CI) using unconditional logistic regression, and meta-analysis when combining these data with our previous report. The association between rs495139 and MOC was not significant in the independent sample (OR = 1.09; 95% CI = 0.97–1.22; *p* = 0.15; N = 665 cases). Meta-analysis suggested a weak association (OR = 1.13; 95% CI = 1.03–1.24; *p* = 0.01; N = 1019 cases). No significant association with risk of other OC histologic types was observed (*p* = 0.05 for tumor heterogeneity). In expression quantitative trait locus (eQTL) analysis, the rs495139 allele was positively associated with ENOSF1 mRNA expression in normal tissues of the gastrointestinal system, particularly esophageal mucosa (*r* = 0.51, *p* = 1.7 × 10^−28^), and nonsignificantly in five MOC tumors. The association results, along with inconclusive tumor eQTL findings, suggest that a true effect of rs495139 might be small.

## 1. Introduction

Ovarian carcinomas of mucinous histology (MOC) are an uncommon type of ovarian cancer characterized by intracellular mucin deposits and relatively favorable prognosis when diagnosed at early stage [1]. Few epidemiologic risk factors are known for these cancers, and the standard risk factors for other types of ovarian cancer do not seem to apply [2]. Increased risk has been associated with current or recent smoking, a higher number of pack years of smoking [3,4], and with increased body mass index [5]. Recently, we reported the first genome-wide significant-susceptibility alleles for MOC at 2q13, 2q31.1, and 19q13.2 [6], and at 3q22.3 and 9q31.1 [7].

Thymidylate synthase (TYMS) is a crucial enzyme for DNA synthesis in both normal and tumor cells. It catalyzes the transformation of dUMP to dTMP and is the only de novo source of thymidylate for pyrimidine biosynthesis [8]. Consequently, it is an important chemotherapy target. We previously found an association between the rs495139 single-nucleotide polymorphism (SNP) and increased risk of MOC (odds ratio (OR) = 1.91; 95% confidence interval (CI) = 1.10–3.31; *p* = 0.02; N = 80 MOC cases) (unpublished) in an earlier genotyping project [9] that we subsequently confirmed (hereto referred to as “discovery” sample) (OR = 1.32; 95% CI = 1.07–1.62; *p* = 0.02; N = 354 MOC cases) [10]. This SNP is located downstream of the 3′ untranslated region (UTR) of *TYMS* (Entrez Gene ID 7298) and situated intronic to the enolase superfamily member 1 (*ENOSF1*, Entrez Gene ID 55556) gene on chromosome 18. *ENOSF1* encodes an antisense transcript that downregulates TYMS gene expression [11,12,13]. We hypothesized that genetic polymorphisms could perturb the TYMS mRNA-antisense mRNA autoregulatory complex by either increasing TYMS or decreasing ENOSF1 gene expression to promote uncontrolled DNA synthesis and tumor growth. Given the SNP’s potential functional role to regulate TYMS expression and because TYMS is an important chemotherapy target, our objective was to replicate the genetic association from our discovery sample [10] using a very large independent sample of MOC from participating studies in the Ovarian Cancer Association Consortium (OCAC) that were genotyped as part of the international Collaborative Oncology Gene-environment Study (iCOGS).

## 2. Results

### 2.1. Association Testing

**Figure 1:**
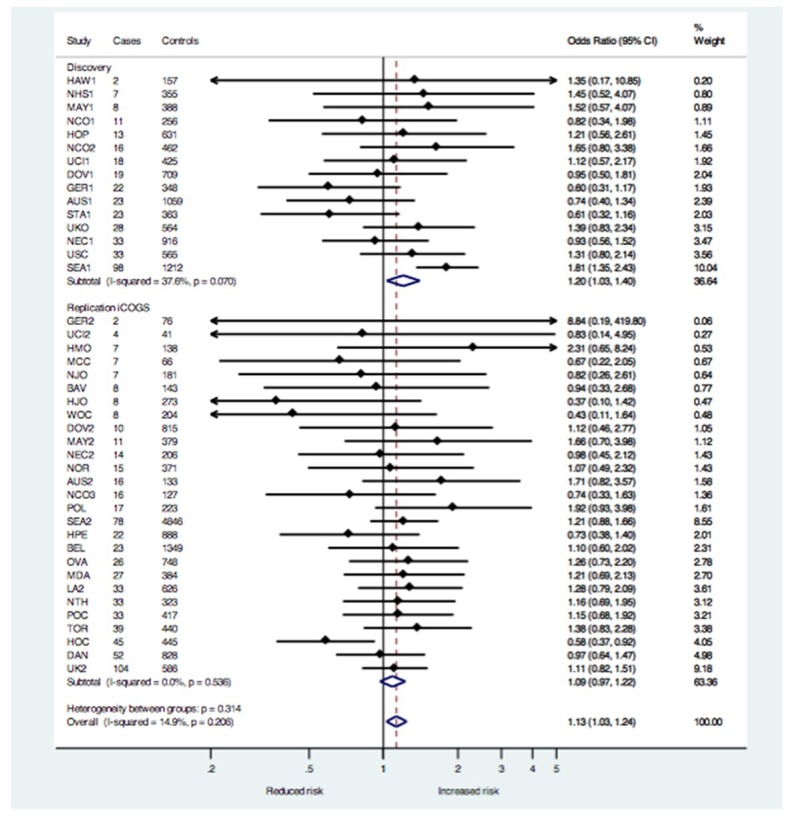
Forest plot of the study-specific (filled diamonds) and pooled (open diamonds) odds ratios and 95% confidence intervals (solid horizontal lines) for the association between rs495139 and risk of MOC under the ordinal genetic risk model in the discovery and iCOGs samples. The total sample size in the meta-analysis is 1019 MOC cases and 23,666 controls of European ancestry. Dashed vertical line represents overall summary estimate–odds ratio. Individual study estimates are adjusted for ancestry and pooled estimates are adjusted for ancestry and study. See Table 1 footnote for details regarding sample size.

There were 667 MOC cases and 15,941 controls in the independent iCOGS sample, but ORs could not be estimated for four individual studies, resulting in 665 MOC cases and 15,256 controls evaluated using the meta-analysis approach. The association between rs495139 and MOC in the iCOGS sample was not significant (OR = 1.09; 95% CI = 0.97–1.22; *p* = 0.15; N = 665 cases) (Table 1). The meta-analysis combining the iCOGS sample with the discovery sample [10] suggested an increased risk (OR = 1.13; 95% CI = 1.03–1.24; *p* = 0.01; N = 1019 cases) (Table 1). The between-group heterogeneity was low to moderate for the discovery studies (*I*^2^ = 37.6), and low for the iCOGS studies (*I*^2^ = 0) and for all studies in the meta-analysis (*I*^2^ = 14.9) (Figure 1). The ORs did not differ appreciably using a pooled-analysis approach that combined all cases and controls from the discovery [10] and iCOGS samples into a single dataset (OR = 1.12; 95% CI = 1.02–1.22; *p* = 0.02; N = 1021 cases) (Table 1). Thus, we proceeded with additional analyses using the pooled data. rs495139 was not associated with ovarian cancer overall or with the other histologic types (Table 1, *p* = 0.05 for tumor heterogeneity, 4 df). Because molecular evidence suggests that many invasive MOC evolve along a multistep model of progression from benign to atypical proliferative (borderline) epithelium [1] similar to colorectal cancer [14], we evaluated the association between rs495139 and borderline MOC only (OR = 0.97; 95% CI = 0.86–1.09; *p* = 0.59, N = 621) and with combined borderline and invasive MOC (OR = 1.06; 95% CI = 0.99–1.14; *p* = 0.11, N = 1642 cases). The weakened associations suggested the SNP might contribute only to the invasive phenotype. Women diagnosed with MOC had an earlier age at diagnosis (median age = 54 years) than women with the more common serous histology (median age = 60 years). To assess whether or not potentially different age distributions between MOC cases and controls influenced ORs, we matched each case to within 5-year age categories to three controls, where possible, and performed an age-stratified logistic regression. The OR was slightly larger (seven cases could not be matched due to young age of diagnosis): OR = 1.16 (95% CI = 1.04–1.28; *p* = 0.006; N = 1013 cases and 3014 controls) when the median age was similar (54 years for cases and 54.7 years for controls).

### 2.2. Expression Quantitative Trait Locus (eQTL) Analysis

We tested for eQTL association between rs495139 genotypes and gene expression in normal tissues in the Genotype Tissue Expression (GTEx) project. The most significant eQTL was with increased ENOSF1 mRNA expression in normal esophagus mucosa (*r* = 0.51, *p* = 1.7 × 10^−28^) (Figures S1 and S2). There was no reported association with TYMS mRNA expression in esophageal mucosa. In 316 women with ovarian cancer (five were MOC tumors), eQTL analysis between germline genotypes of rs495139 and tumor gene expression showed positive associations for two probes targeting ENOSF1 (P15906: beta = 0.17, *p* = 0.03, and P4503: beta = 0.15, *p* = 0.05) but not with the probe targeting TYMS (P50096: beta = 0.12, *p* = 0.12) (Table S1). Among the five MOC tumors, we observed positive eQTL associations with one probe targeting ENOSF1 (P4503: beta = 0.87, *p* = 0.32) but not the other (P15906: beta = 0.08, *p* = 0.94) and an inverse eQTL association with the probe targeting TYMS (P50096: beta = −0.70, *p* = 0.45) but none were statistically significant given the small sample size.

## 3. Discussion

We previously observed a statistically significant risk association of rs495139 [10], which was not replicated by this larger analysis of MOC. Combining the two independent datasets of 1019 MOC samples suggested a weakly positive association between rs495139 and risk of MOC, particularly in age-matched analysis. The association observed from the combined analysis was attenuated with the inclusion of borderline cases.

Few genetic risk factors for MOC are known. We previously published results from two genome-wide association studies (GWAS) reporting five susceptibility variants for MOC at 2q13, 2q31.1, 19q13.2 [6], 3q22.3, and 9q31.1 [7]. The likelihood that the candidate SNP rs495139 is a genetic risk factor for MOC is small. Since the time of preparing this manuscript, we conducted a third, larger GWAS of ovarian cancer [7], which included many of the participants in the current report. Results from that GWAS also do not support significant associations between rs495139 and invasive MOC (OR = 0.93; 95% CI = 0.86–1.01; *p* = 0.07, 1417 cases) or combined borderline and invasive MOC (OR = 0.97; 95% CI = 0.91–1.03; *p* = 0.29, 2566 cases). Our results suggest the previously published significant association [10] was likely driven by heterogeneity from a few studies with extreme ORs and that balanced age matching between MOC cases and controls might reduce some bias. A strong functional influence of rs495139 on increased ENOSF1 gene expression was found in GTEx normal gastrointestinal tissues and, in particular, esophageal mucosa. This is interesting because the origin of normal tissue from which MOC arises is unclear but may be similar to the mucosa of the gastrointestinal system [1,15]. For example, small array-based studies (N = 3 to 9) of MOC relate them more closely to colonic epithelium or colorectal cancers than to normal ovarian surface epithelium [15,16]. Further, some MOC may arise from a morphologically analogous transition observed in gastroesophageal adenocarcinoma. The metaplasia seen in MOC is a change in cell type from a monolayer of normal nondescript, poorly differentiated mesothelial cells [17] to a single layer of tall, columnar epithelial cells with mucin-containing cytoplasm [18]. This is analogous to the metaplasia seen in the development of Barrett’s esophagus, the precursor lesion to gastroesophageal adenocarcinoma, where there is a change in cell type from a flat squamous cell layer to columnar-like epithelium with visible mucus [19]. We also observed a nonsignificant but positive eQTL association between rs495139 and a probe for ENOSF1 mRNA in MOC, as well as an inverse eQTL association between rs495139 and TYMS mRNA in MOC tumors, which contradicts our hypothesis that rs495139 might decrease ENOSF1 antisense mRNA to possibly increase TYMS mRNA availability for DNA synthesis and tumor growth. The small number of MOC tumors in this eQTL analysis precluded any definitive interpretation of results. Given that TYMS is a chemotherapy target, an rs495139-associated increase in ENOSF1 and decrease in TYMS expression might suggest a favorable tumor profile for patient survival.

This investigation was strengthened by including large numbers of women with MOC that contributed individual genotype data in iCOGS and by the ability to conduct an age-matched analysis to control for differences in the distribution of age among cases and controls, which could potentially bias associations. A further advantage was our centralized genotyping and rigorous quality-control standards. We were also able to query data in silico as well as in women with corresponding germline SNP and tumor gene expression data from the Mayo Clinic in order to provide contextual information on potential functional influences between genotypes and tumor gene expression.

In summary, the evidence in this study to support a role of rs495139 in the *TYMS-ENOSF1* region as a genetic risk factor for MOC is weak and suggests that any true effect is likely to be small.

## 4. Materials and Methods

### 4.1. Study Subjects and Genotyping

Subjects (*n* = 47,630) represented multiple individual studies participating in the OCAC [20]. Informed consent was obtained in individual studies and local human research investigation committees approved each study. *TYMS-ENOSF1* rs495139 was genotyped on an Illumina Infinium custom iSelect BeadChip developed for the iCOGS (Beadchip) [21]. Among 44,634 subjects whose DNA passed centralized genotyping quality-control criteria [20], the call rate for rs495139 was 99.98%. We restricted the analysis to subjects with European ancestry and invasive tumors, leaving 37,792 eligible subjects. Of these, the effect allele frequency (EAF) among 23,444 controls was 0.41 and no departure from Hardy–Weinberg equilibrium was seen (*p* = 0.32).

To obtain the independent sample (referred to as the “iCOGS” sample), we excluded genotypes in iCOGS from women who were in our earlier analysis [10] (referred to as the “discovery” sample). Individual-level genotypes were available on 10,019 invasive ovarian cancer cases (667 with invasive MOC) and 15,941 controls from 31 studies in iCOGS for replication analysis. We also re-evaluated the rs495139 association by combining genotypes from the iCOGS sample with the discovery sample (4981 invasive ovarian cancer cases, of which 354 were invasive MOC, and 8410 controls from 14 OCAC studies) [10], resulting in a total of 15,000 invasive ovarian cancer cases (1021 with invasive MOC) and 24,351 controls (Table S2). This larger sample allowed additional subset analyses to be performed.

### 4.2. In Silico and eQTL Analysis

We used the GTEx project portal (V7 data release, 18 September 2017) [22] for in silico eQTL analysis between rs495139 and TYMS and ENOSF1 gene expression in 10,294 eQTL normal tissues representing over 50 different tissue types. We evaluated eQTL between rs495139 genotypes from lymphocytes with gene expression from patient-matched tumors among 316 women with ovarian cancer (including five MOC tumors) from the Mayo Clinic. Gene expression was assessed using a 4 × 44 K Agilent array (Agilent Technologies, Santa Clara, CA, USA) and measured as log_2_(tumor/reference) probe intensity signals as described previously [23] based on a mixed tumor cell-type reference of 104 tumor samples, including papillary serous (*n* = 67), endometrioid (*n* = 5), mucinous (*n* = 3), clear cell (*n* = 3), malignant mixed Müllerian tumor (*n* = 5), goblet cell (*n* = 1), squamous (*n* = 1), transitional cell (*n* = 1), benign (*n* = 4), normal (*n* = 7), and unknown epithelial (*n* = 7). Analyses included one Agilent probe for TYMS (P50096) and two probes for ENOSF1 (P15906 and P4503).

### 4.3. Statistical Analysis

ORs and 95% CIs were estimated using unconditional logistic regression assuming an ordinal (log-additive) genetic model and adjusting for study and for the first five eigenvalues from principal components analysis to account for substrata of European ancestry [20]. Using fixed-effects meta-analysis, we combined study-specific effect estimates from our previous report [10] with study-specific effect estimates from unique iCOGS subjects using the inverse-variance method to weight the overall summary OR. The degree of statistical heterogeneity between studies was estimated by *I*^2^, the between-group variance [24]. Studies with statistically homogeneous ORs yield an *I*^2^ value of zero. ORs were derived for ovarian cancers overall and simultaneously for the different histologic types (serous, mucinous, endometrioid, and clear cell) using polytomous logistic regression. Statistical heterogeneity of the SNP-ovarian carcinoma histology associations was tested using the type 3 analysis of effects with 4 degrees of freedom [25]. Van der Waerden rank transformation was applied to tumor gene expression values from the Agilent array, adjusting for age in linear regression and treating the number of variant alleles carried as ordinal on the log-additive scale. Statistical tests were two-sided and implemented with SAS version 9 (SAS Institute, Cary, NC, USA). Meta-analysis was performed with Stata/SE (version 13.1, StataCorp, College Station, TX, USA).

## Figures and Tables

**Table 1:** Associations ^1^ between rs495139 and ovarian carcinoma among European subjects.

Study Sample and Tumor Histology	Analysis Method	Cases, N	Controls, N	OR	95% CI	*p*-Value
**iCOGS sample**						
Mucinous invasive only	Meta-analysis	665 ^2^	15,256 ^2^	1.09	0.97–1.22	0.16
**Discovery + iCOGS samples**						
Mucinous invasive only	Meta-analysis	1019 ^2^	23,666 ^2^	1.13	1.03–1.24	0.01
All invasive tumors ^3^	Pooled	15,000	24,351	1.00	0.97–1.03	0.84
Mucinous invasive only	Pooled	1021 ^2^	24,351	1.12	1.02–1.22	0.02
Mucinous borderline	Pooled	621	24,351	0.97	0.86–1.09	0.59
Mucinous invasive and borderline combined	Pooled	1642	24,351	1.06	0.99–1.14	0.11
Serous invasive	Pooled	8889	24,351	1.01	0.98–1.05	0.53
Endometrioid invasive	Pooled	2164	24,351	0.97	0.91–1.04	0.40
Clear cell invasive	Pooled	1046	24,351	0.93	0.85–1.02	0.11

iCOGS, International Collaborative Oncology Gene-environment Study. ^1^ Studies in the meta-analysis were restricted to non-Hispanic whites (Discovery set), as in the original report [10], or adjusted for European ancestry using principal components analysis (iCOGS). Studies in the pooled analysis were also adjusted for study set. ^2^ Two studies (HAW and STA) in iCOGS each contributed one mucinous ovarian carcinoma (MOC) case and two studies (NHS and MSK) did not contribute any MOC cases in iCOGS; therefore, odds ratios could not be calculated for these four studies and these subjects (HAW: 1 MOC case, 17 controls; STA: 1 MOC case, 6 controls; NHS: 0 MOC cases, 69 controls; MSK: 0 MOC cases, 593 controls) were dropped from the meta-analysis but were retained in the pooled analysis. ^3^ Includes invasive tumors with the following histology: serous; mucinous; endometrioid; clear cell; mixed cell; other specified epithelial ovarian cancer; undifferentiated or poorly differentiated epithelial; and unknown histology but known to be epithelial.

## Supplementary Materials

Supplementary materials can be found at http://www.mdpi.com/1422-0067/19/9/2473/s1.

## Abbreviations

dTMP	Deoxythymidine monophosphate
dUMP	Deoxyuridine monophosphate
ENOSF1	Enolase superfamily member 1
eQTL	Expression Quantitative Trait Locus
GTEx	Genotype Tissue Expression project
iCOGS	international Collaborative Oncology Gene-environment Study
MOC	Mucinous ovarian carcinoma
mRNA	Messenger ribonucleic acid
OCAC	Ovarian Cancer Association Consortium
SNP	Single nucleotide polymorphism
TYMS	Thymidylate synthase
UTR	Untranslated region
