# Supplementary material for: rs495139 in the TYMS-ENOSF1 Region and Risk of Ovarian Carcinoma of Mucinous Histology
Source: Int J Mol Sci. 2018 Aug 21;19(9):2473. doi: 10.3390/ijms19092473 (PMC6163881; doi:10.3390/ijms19092473)
Supplement: Supplementary file 1 [file ijms-19-02473-s001.zip › ijms-330135-SI.pdf]

Supplemental Table 1. Age-adjusted expression quantitative trait locus associations between rs495139 and TYMS and ENOSF1 gene expression in 316 ovarian tumors, Mayo Clinic.

			**All tumors, N=316**		**MOC, N=5**	
**Agilent Probe**	**Coordinates (Chr 18)***	**Gene region**	**Beta**	**P-value**	**Beta**	**P-value**
A-23_P50096	673276-673335	TYMS	0.12	0.12	-0.70	0.45
A_23_P15906	674217-674158	ENOSF1	0.17	0.03	0.08	0.94
A-23_P4503	688576-685952	ENOSF1	0.15	0.05	0.87	0.32

* Human genome reference build 37

Supplemental Table 2. Contributing studies of invasive ovarian cancer cases and controls of European ancestry, OCAC[a]

OCAC Study Name	Study Abbrev.	Analysis Set	Study Location	Study Design	Controls	All cases	Mucinous cases
**Discovery set**							
Australian Ovarian Cancer Study	AOC	AUS	Australia	Population-based/ case control	1059	717	23
Diseases of the Ovary and their Evaluation	DOV	DOV	USA: 13 counties in western Washington state	Population-based/ case control	709	526	19
German Ovarian Cancer Study	GER	GER	Germany: two geographical regions in the states of Baden-Württemberg and Rhineland-Palatinate in southern Germany	Population-based/ case control	348	197	22
Hawaii Ovarian Cancer Case-Control Study	HAW	HAW	USA: Hawaii	Population-based/ case control	157	70	2
Novel Risk Factors and Potential Early Detection Markers for Ovarian Cancer	HOP	HOP	USA: western PA, northeastern Ohio, western New York	Population-based/ case control	631	278	13
Mayo Clinic Ovarian Cancer Case-Control Study	MAY	MAY	USA: North-Central (MN, SD, ND, IL, IA, WI)	Clinic-based/ case control	388	303	8
North Carolina Ovarian Cancer Study	NCO	NCO	USA: central and eastern North Carolina (48 counties)	Population-based/ case control	718	567	27
New England Case Control Study	NEC	NEC	USA: New Hampshire and eastern Massachusetts	Population-based/ case control	916	512	33
Nurses' Health Study	NHS	NHS	USA: National	Population-based/ Nested case control	355	109	7
Study of Epidemiology and Risk Factors in Cancer Heredity	SEA	SEA	UK: East Anglia and West Midlands	Population-based/ case control	1,212	523	98
Family Registry for Ovarian Cancer and Genetic Epidemiology of Ovarian Cancer	STA	STA	USA: Six counties in the San Francisco Bay area	Population-based/ Case control	363	247	23
University of California Irvine Ovarian Study	UCI	UCI	USA: Southern California (Orange and San-Diego, Imperial Counties)	Population-based/ Case control	425	284	18
United Kingdom Ovarian Cancer Population Study	UKO	UKO	UK: England, Wales and Northern Ireland	Population-based/ Case control	564	244	28
Los Angeles County Case-Control studies of Ovarian Cancer-1	USC	USC	USA: Los Angeles County, California	Population-based/ Case control	565	404	33
**Total**					**8,410**	**4,981**	**354**

OCAC Study Name	Study Abbrev.	Analysis Set	Study Location	Study Design	Controls	All cases	Mucinous cases
**iCOGS set**							
Australian Cancer Study (Ovarian Cancer)	ACS	AUS	Australia	Population-based/ case control	28	31	2
Australian Ovarian Cancer Study	AOC	AUS	Australia	Population-based/ case control	105	272	14
Bavarian Ovarian Cancer Cases and Controls	BAV	BAV	Southeast Germany	Population-based/ case control	143	93	8
Belgian Ovarian Cancer Study	BEL	BEL	Belgium, University Hospital Leuven	Hospital based/ Case control	1,349	269	23
Danish Pelvic Mass Study	PVD	DAN	Denmark	Case only	0	169	10
Malignant Ovarian Cancer	MAL	DAN	Denmark	Population-based/ Case control	828	440	42
Diseases of the Ovary and their Evaluation	DOV	DOV	USA: 13 counties in western Washington state	Population-based/ case control	815	405	8
Oregon Ovarian Cancer Registry	ORE	DOV	USA: Portland, Oregon	Case only	0	55	2
German Ovarian Cancer Study	GER	GER	Germany: two geographical regions in the states of Baden-Württemberg and Rhineland-Palatinate in southern Germany	Population-based/ case control	76	8	1
Dr. Horst Schmidt Kliniken	HSK	GER	Germany	Case only	0	144	1
Hawaii Ovarian Cancer Case-Control Study	HAW	HAW	USA: Hawaii	Population-based/ case control	17	9	1
Hannover-Jena Ovarian Cancer Study	HJO	HJO	Germany	Hospital based/ Case control	273	255	8
Hannover-Minsk Ovarian Cancer Study	HMO	HMO	Belarus	Case control	138	143	7
Helsinki Ovarian Cancer Study	HOC	HOC	Helsinki, Finland	Case control	445	213	45
Gilda Radner Familial Ovarian Cancer Registry	GRR	HPE	USA: National	Familial cancer/ Case only	0	110	5
Novel Risk Factors and Potential Early Detection Markers for Ovarian Cancer	HOP	HPE	USA: Western PA, Northeastern Ohio, Western New York	Population-based/ Case control	888	374	17
Women's Cancer Program	LAX	LA2	USA: Southern California	Case only	0	272	12

OCAC Study Name	Study Abbrev.	Analysis Set	Study Location	Study Design	Controls	All cases	Mucinous cases
at the Samuel Oschin Comprehensive Cancer Institute							
Los Angeles County Case-control studies of Ovarian Cancer-1	USC	LA2	USA: Los Angeles County, California	Population-based/ Case control	626	394	21
Mayo Clinic Ovarian Cancer Case-Control Study	MAY	MAY	USA: North Central (MN, SD, ND, IL, IA, WI)	Clinic based/ Case control	379	414	11
Melbourne Collaborative Cohort Study	MCC	MCC	Melbourne, Australia	Cohort/ Nested case control	66	63	7
MD Anderson Ovarian Cancer Study	MDA	MDA	USA: Texas	Hospital based/ Case control	384	313	27
Memorial Sloan–Kettering Cancer Center	MSK	MSK	USA: New York City	Case control	593	454	0
North Carolina Ovarian Cancer Study	NCO	NCO	USA: Central and eastern North Carolina (48 counties)	Population-based/ Case control	127	214	16
New England Case-Control Study	NEC	NEC	USA: New Hampshire and Eastern Massachusetts	Population-based/ Case control	206	226	14
Nurses' Health Study	NHS	NHS	USA: National	Population-based/ Nested case control	69	16	0
New Jersey Ovarian Cancer Study	NJO	NJO	USA: New Jersey (six counties)	Case control	181	168	7
University of Bergen, Haukeland University Hospital, Norway	NOR	NOR	Norway	Case control	371	227	15
Nijmegen Ovarian Cancer Study	NTH	NTH	Eastern part of the Netherlands	Case control	323	255	33
Ovarian Cancer in Alberta and British Columbia	OVA	OVA	Alberta and British Columbia, Canada	Case control	748	605	26
Polish Ovarian Cancer Study	POC	POC	Poland: Szczecin, Poznan, Opole, Rzeszów	Case control	417	421	33
Polish Ovarian Cancer Case-Control Study	POL	POL	Poland: Warsaw, and Lodz	Population-based/ Case control	223	236	17
Study of Epidemiology and Risk Factors in Cancer Heredity	SEA	SEA	UK: East Anglia and West Midlands	Population-based/ Case control	4,846	808	78
Family Registry for Ovarian Cancer and Genetic Epidemiology of Ovarian Cancer	STA	STA	USA: Six counties in the San Francisco Bay area	Population-based/ Case control	6	40	1
Toronto Ovarian Cancer Study	TOR	TOR	Canada: Province of Ontario	Population-based	440	557	39
University of California	UCI	UCI	USA: Southern California	Population-	41	67	4

OCAC Study Name	Study Abbrev.	Analysis Set	Study Location	Study Design	Controls	All cases	Mucinous cases
Irvine Ovarian Study			(Orange and San-Diego, Imperial Counties)	based/ Case control			
Royal Marsden Hospital Ovarian Cancer Study	RMH	UK2	UK: London	Hospital-based/ case only	0	145	16
Southampton Ovarian Cancer Study	SOC	UK2	UK: Wessex region	Case only/ hospital-based	0	266	33
Scottish Randomised Trial in Ovarian Cancer	SRO	UK2	UK: coordinated through clinical trials unit, Glasgow UK from patients recruited worldwide	Case only/ clinical trial	0	152	3
United Kingdom Ovarian Cancer Population Study	UKO	UK2	UK: England, Wales, and Northern Ireland	Population-based/ Case control	586	470	49
UK Familial Ovarian Cancer Registry	UKR	UK2	UK: National	Case only/ familial register	0	47	3
Warsaw Ovarian Cancer Study	WOC	WOC	Poland: Warsaw and central Poland	Case control	204	200	8
**Total**					**15,941**	**10,019**	**667**
**Overall Total**					**24,351**	**15,000**	**1,021**

[a]Ovarian Cancer Association Consortium

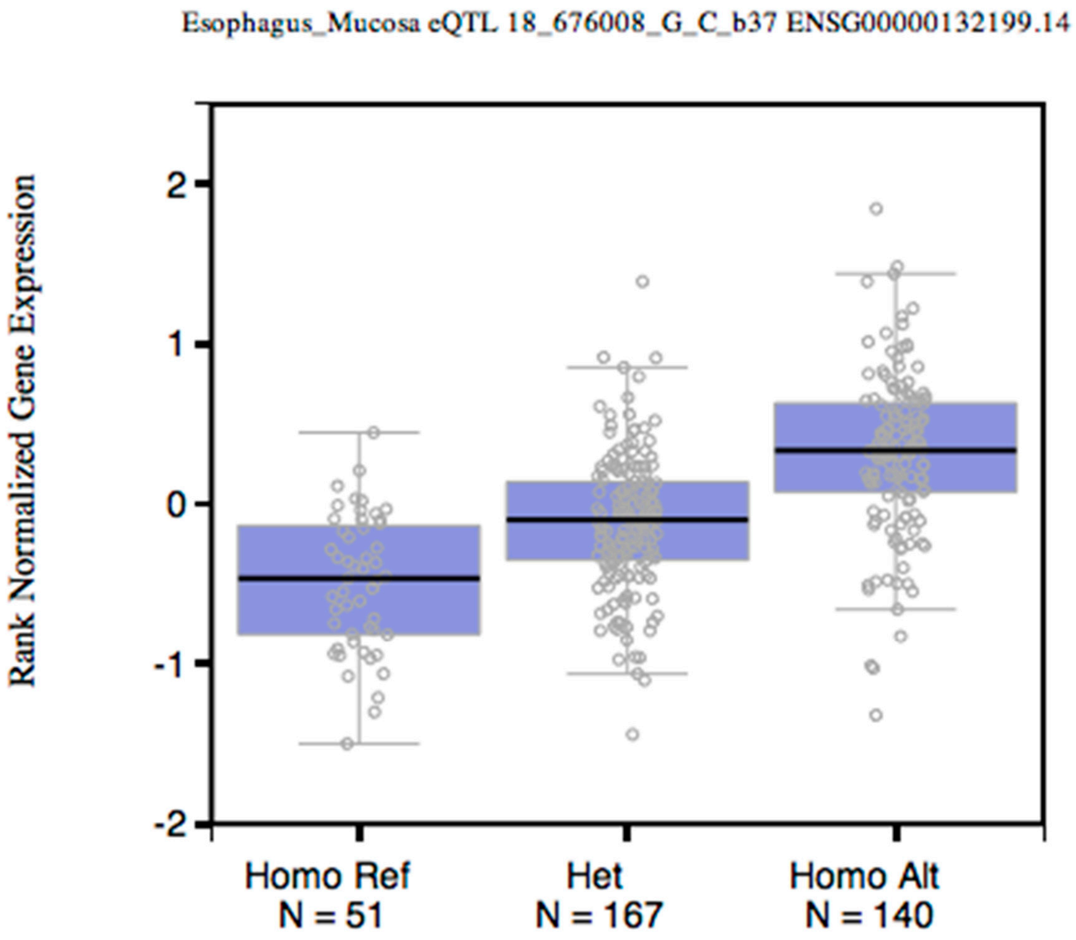

Supplemental Figure 1. Correlation between rs495139 and ENOSF1 mRNA expression in normal esophageal mucosa from the Genotype-Tissue Expression project.

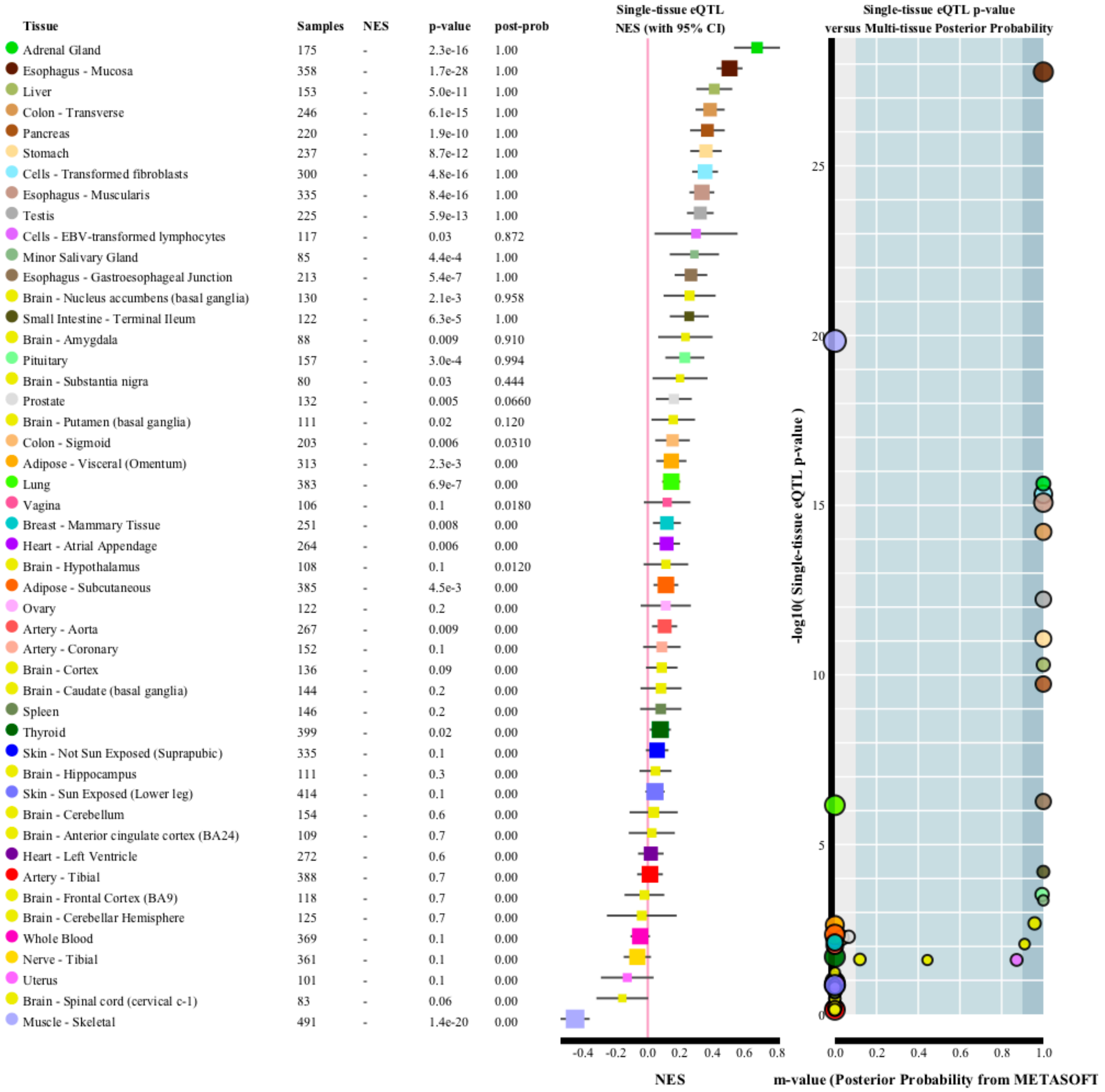

Supplemental Figure 2. P values for expression quantitative trait locus analysis between rs495139 and ENOSF1 mRNA expression in different tissues in the Genotype-Tissue Expression project.
